# Impact of Infectious Diseases training in the perception of antibiotic resistance and rational use of antibiotics among Spanish medical students – a cross-sectional study

**DOI:** 10.1186/s12909-022-03580-8

**Published:** 2022-07-15

**Authors:** José Ramón Yuste, Andrés Blanco-Di Matteo, Fernando Gruber

**Affiliations:** 1 Division of Infectious Diseases, Faculty of Medicine, University of Navarra, Clinica Universidad de Navarra, Pamplona, Spain; 2 Department of Internal Medicine, Faculty of Medicine, University of Navarra, Clinica Universidad de Navarra, Pamplona, Spain

**Keywords:** Antibiotics, Antimicrobial resistance, Clinical education, Medical training

## Abstract

**Background:**

Antibiotic resistance is one of the main public health problems worldwide. One key tool to optimize antibiotic prescription is medical training. The aim of this study is to compare the impact of training in infectious diseases on students’ knowledge of the antibiotic resistance problem and the rational use of antibiotics.

**Methods:**

We performed a cross-sectional study in the medical school of the University of Navarra. We conducted an anonymous in situ survey of students in each year of training. Data were analyzed grouping the students as follows: GROUP 1: first three years of education, no training in Clinical Microbiology (CM) or in Infectious Diseases (ID); GROUP 2: fourth-year students, training in CM but not ID; GROUP 3: Fifth and sixth-year students who have completed the training in CM and ID. Chi-square test (or Fisher’s exact test when appropriate) was performed to evaluate potential associations. Wilcoxon’s test was used to compare the median correct answers between groups. We used Spearman’s test for correlation between year of training and performance in questionnaire.

**Results:**

A total of 994 students respond to the survey, 80.4% of the eligible students. Almost all students who had completed infectious diseases training perceive antibiotic resistance as an important problem in comparison with students who had not completed the formation (99.5% in group 3 vs 94.5% in group 1, *p* = 0.02). Knowledge of antibiotic stewardship underwent a statistically significant change after training in infectious diseases (from 9.2% in group 1 to 52.2% in group 3, *p* < 0.001). In the training questions block we also found an increase in the average number of correct answers (21.4% in group 1 vs 44.7% in group 3, *p* < 0.001). When comparing the results of subgroups 3A and 3B we found a significant loss of knowledge as we moved away from training (49% vs 40.9%, *p* < 0.001).

**Conclusions:**

The training of medical students is the key to improving both perception and knowledge of infectious diseases. However, we have an opportunity for educational improvement as far as infectious diseases are concerned, regarding both the acquisition of knowledge and its loss as time lapses after training.

**Supplementary Information:**

The online version contains supplementary material available at 10.1186/s12909-022-03580-8.

## Background

According to the World Health Organization, the antibiotic resistance crisis is one of the main public health problems worldwide [1, 2]. Antibiotic resistance is rising to dangerously high levels in all parts of the world. New resistance mechanisms are emerging and spreading globally, threatening our ability to treat common infectious diseases. This problem has been attributed to several aspects, including misuse of antibiotics, use without prescription, used by veterinarians, overuses of long therapies and lack of knowledge of resistance mechanisms. Infections by resistant bacteria cannot be treated with standard antibiotic treatment, the lack of activity increase number of morbidity and mortality cases and increase healthcare expense. But some authors have shown that general practitioners consider antibiotic resistance to be a rare issue and may misinterpret the evidence that links inappropriate antibiotic prescription with bacterial resistance [3]. The recognition of antibiotic resistance as public health problem is mandatory, and basic infectious diseases training in medical school is an excellent opportunity to achieve this need.

Several studies show that in 30–50% of cases in which antibiotics are prescribed, their use can be optimized. The most common misuse are unnecessary prolonged therapy, antibiotic coverage too broad in empirical therapy, delay in the change of administration route or de-escalation. Healthcare education and training for medical students on rational antimicrobial prescribing or antimicrobial stewardship are a key tool to contain antimicrobial resistance [4, 5]. To date, investigation of students attitudes towards antibiotic prescription and perceptions of antibiotic resistance has focused on final-year medical students [6–8]. We thought that comparing students before and after receiving training in infectious diseases could give us fundamental information to understand the learning process and establish which concepts, we need to emphasize.

The aim of this study is to compare the impact of training in infectious diseases in the knowledge of the antibiotic resistance problem and the rational use of antibiotics.

## Methods

### Participants

We conducted a cross-sectional study including all medical students at the medical school of the University of Navarra. The University of Navarra is one of the top private universities in Spain. It is located in Pamplona and was ranked 266th in the QS Global World Rankings in 2022.

In September 2018, at the beginning of the academic year, there were a total of 1236 students: 214 in the first year, 199 in the second year, 193 in the third year, 222 in the fourth year, 204 in the fifth year and 204 in the sixth year. In the University of Navarra, the training in clinical microbiology is carried out during the third year and infectious diseases education in the fourth year. Students who complete the six-year program receive a medical degree and the authorization to prescribe antibiotics. The survey was conducted between 17 September and 21 September 2018.

**Fig. 1:**
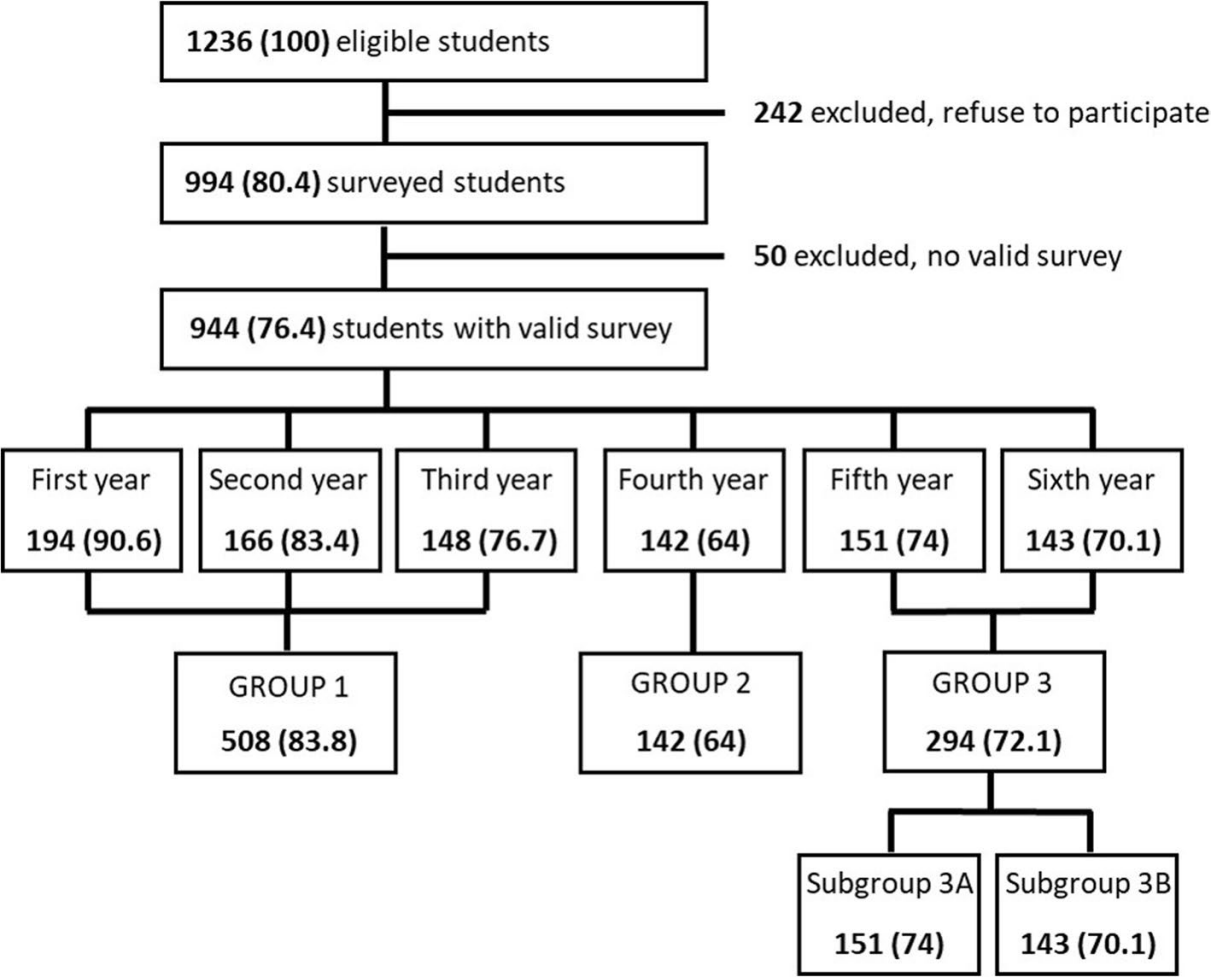
Division of students according to year of training and time of survey

To simplify the analyses, we divided subjects into 3 groups and 2 subgroups. GROUP 1 is made up untrained students in the first, second and third year of medical school. Students who completed training in Clinical Microbiology but not Infectious Diseases formed GROUP 2 (fourth year students). Fully trained students in Clinical Microbiology and Infectious diseases formed GROUP 3 (fifth and sixth year). We divided group 3 in two subgroups according to the time that had elapsed from completed training. In SUBGROUP 3A they completed training in Infectious Diseases in the same year as the survey (fifth year students) and in the SUBGROUP 3B they had completed training in Infectious Diseases one year before the survey (sixth year students). See Fig. 1 for a graphic representation of the division of groups.

### Preparation of the survey

We developed a questionnaire with 19 questions (see Online Resource 1). The survey was divided into 2 blocks: the first block was intended to understand the students’ knowledge about diagnosis, bacterial resistance, and appropriate use of antibiotics, and the second block to evaluate their expertise in infectious diseases.

The first block included 12 questions with a Likert-type scale with 4 possible answers: always, frequently, rarely, and never. To facilitate the analysis, options always and frequently were grouped as “Positive” and rarely or never as “Negative”. The second block included 7 questions; six of them had 4 distractors with only one correct answer. The questionnaire was approved by the dean of the medical school. The ethics committee approved the study.

Participation was voluntary, anonymous and without compensation. The survey was conducted in the last 15 minutes of a class in which most students from each year of were present. The attendance list was used to verify that all the subjects present in the classroom belonged to the respective year being to be evaluated. The Socrative online educational platform was used (available at www.socrative.com), and each student used their personal mobile device to complete it. The surveys were identified with the year of training.

Questions in the first block assessed the magnitude of antimicrobial resistance; the importance of collecting samples for microbiological cultures; the indications for initiation of empirical antibiotic treatment; the value of microbiological information and clinical evolution for the de-escalation therapy; the usefulness of combination therapy; the duration of antibiotic therapy; the route of administration; the rational use of restricted antibiotics; the antibiotic cost assessment and the knowledge about antibiotic stewardship programs. Questions in the second block were oriented to evaluate the knowledge of basic infectious disease scenarios such as microorganism associated with multidrug resistance, appropriate media cultures, best antibiotic empirical treatment, the use of antibiotics in non-bacterial infection, treatment of asymptomatic bacteriuria, route of treatment and length of therapy.

To consider a survey as valid it had to have at least 5 questions answered. Surveys that did not meet this criterion were not analyzed in this study.

### Data analysis

All analyses were performed on a per protocol basis using SPSS software (version 25.0). Chi-square test (or Fisher’s exact test when appropriate) was performed to evaluate potential associations. Wilcoxon’s test was used to compare the median correct answers between groups. We used Spearman’s test for correlation between year of training and performance in questionnaire. *P*-values < 0.05 were considered statistically significant.

## Results

**Fig. 2:**
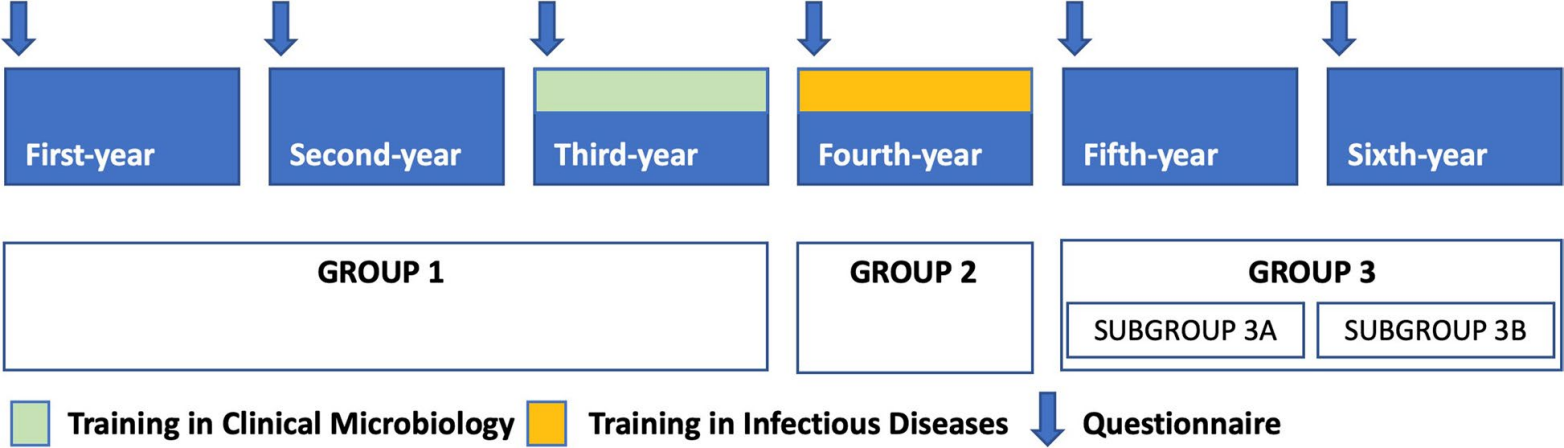
Flow chart of participation and group allocation. Data expressed in numbers (%)

Out of 1236 eligible medical students, 994 (80.4%) responded to the survey with 944 (76.4%) valid surveys. Table 1 shows the students surveyed by year and group of training. Figure 2 shows a flow chart summarizing participation and group allocation.

**Table 1:** Proportion of students surveyed by year and group of training

	*Total students* *n*	*Students surveyed* *n (%)*	*Not valid surveys* *n (%)*	*Valid* *surveys* *n (%)*
***Year of training***				
First	214	198 (92.5)	4 (1.9)	194 (90.6)
Second	199	178 (89.4)	12 (6.0)	166 (83.4)
Third	193	158 (81.9)	10 (5.2)	148 (76.7)
Fourth	222	150 (67.6)	8 (3.6)	142 (64.0)
Fifth	204	155 (76.0)	4 (2.0)	151 (74.0)
Sixth	204	155 (76.0)	12 (5.9)	143 (70.1)
***Group of training***				
Group 1	606	534 (88.1)	26 (4.3)	508 (83.8)
Group 2	222	150 (67.6)	8 (3.6)	142 (64.0)
Group 3	408	310 (76.0)	16 (3.9)	294 (72.1)
Subgroup 3A	204	155 (76.0)	4 (2.0)	151 (74.0)
Subgroup 3B	204	155 (76.0)	12(5.9)	143 (70.1)
***Total***				
	*1236*	*994 (80.4)*	*50 (4.0)*	*944 (76.4)*

### Students’ knowledge

Table 2 presents an overview of the students’ knowledge by training. Comparing responses of students from group 3 and group 1, 99.3% of students considered that antibiotic resistance always/frequently represents a Public Health problem and 94.7% in group 1 thought of it as a problem (*p* = 0.02), while 98.4% in group 3 believed antibiotic resistance should be always or frequently considered before starting an empirical antibiotic treatment, compared with 96.4% in group 1 (*p* = 0.17).

**Table 2:** Comparison between answers of the students without training (Group 1) and the trained students in infectious diseases (Group 3)

	*Group 1%*	*Group 3%*	*p*
***Relevance of the problem***			
*Question 1*. Do you think antibiotic resistance is a public health problem?			
Always-Frequently	94.7	99.3	*p = 0.02*
Rarely-Never	5.3	0.7	
***Microbiological cultures***			
*Question 2.* Do you think collecting specimens for microbiological cultures before starting empirical antibiotics is mandatory?			
Always-Frequently	68.6	90.3	*p < 0.001*
Rarely-Never	31.4	9.7	
***Empirical antibiotic treatment***			
*Question 3.* In a clinically stable patient with documented clinical infection, is it necessary to start empirical antibiotic treatment?			
Always-Frequently	78.3	75.7	*p* = 0.37
Rarely-Never	21.7	24.4	
*Question 4.* Before starting empirical antibiotic treatment, should we consider the problem of antibiotic resistance?			
Always-Frequently	96.4	98.4	*p* = 0.17
Rarely-Never	3.6	1.6	
***De-escalation therapy***			
*Question 5.* Do you think empirical antibiotic treatment should be adjusted according to microbiological data and clinical evolution?			
Always-Frequently	96.9	97.8	*p* = 0.63
Rarely-Never	3.1	2.2	
***Combination therapy***			
*Question 6.* Do you think antibiotic combinations improve clinical results?			
Always-Frequently	93.5	85.3	*p* = 0.61
Rarely-Never	6.5	14.7	
***Prolonged duration***			
*Question 7*. Do you think prolonged duration of antibiotic treatment beyond what is recommended by clinical guidelines improves clinical outcomes?			
Always-Frequently	23.2	9.5	*p < 0.001*
Rarely-Never	76.8	90.5	
***Appropriate route***			
*Question 8*. Should conversion from parenteral to oral therapy of antibiotics with excellent bioavailability in clinically stable infected be considered?			
Always-Frequently	48.3	84.2	
Rarely-Never	51.7	15.8	*p < 0.001*
***Antibiotics for restricted use***			
*Question 9*. Do you think use of restricted drugs provides a clinical and microbiological benefit over first-line drugs?			
Always-Frequently	65	45.9	*p < 0.001*
Rarely-Never	35	54.1	
***Antibiotic cost***			
*Question 10*. Do you think cost of antibiotic treatment should be considered before its prescription?			
Always-Frequently	67	85.4	*p < 0.001*
Rarely-Never	33	14.6	
***Antibiotic stewardship program***			
*Question 11*. Do you know the Antibiotic Stewardship Program?			
Yes	9.3	52.2	*p < 0.001*
No	90.7	47.8	
*Question 12*. Do you think the antibiotic Stewardship Program can improve medical training and clinical outcomes in the hospitals in which it is established?			
Always-Frequently	99	99.1	*p* = 0.97
Rarely-Never	1	0.9	

In empirical antibiotic treatment, 78.3% of group 3 students assumed that antibiotic therapy always/frequently should be started in a stable clinical infected patient compared to 75.7% in group 1 (*p* = 0.37). As for fully trained students, 90.3% of them believed that prior microbiological sample collection always or frequently should be performed, compared with 68.6% of students in the group who had not received microbiology or infectious disease formation so far (*p* < 0.001).

In adjusted antibiotic treatment, de-escalation therapy was always/frequently considered by 98.4% of students in group 3 and 96.4% in group 1 (*p* = 0.63). 84.2% of fully trained students always or frequently considered conversion from parenteral to oral treatment when available, compared with just 48.3% of students in group 1 (*p* < 0.001). Regarding combination therapy, no statistically significant differences were detected, because 85.3% of students in group 3 always or frequently considered combination therapy to be useful and 93.5% of students in group 1 had the same perception (*p* = 0.61). There was a statistically significant difference between groups in the perception of the use of restricted drugs for improving clinical and microbiological results (45.9% in group 3 vs 65% in group 1; *p* < 0.001). Just 9.5% of group 3 students always/frequently evaluated prolonged therapy improve clinical outcomes compared with 23.2% of students in group 1 (*p* < 0.001).

In group 3, 52.2% of students were familiar with antibiotic stewardship programs compared with 9.3% students in group 1 with a statistically significant difference (*p* < 0.001).

### Impact of undergraduate training in infectious diseases

The percentage of correct answers was 25.3% in group 1, 32.4% in group 2 (after training in clinical microbiology), and 52.7% in group 3 (after training in infectious diseases). The median number of correct answers for students in group 1 was 1.60 [95% CI 1.50–1.70], in group 2 it was 1.93 [95% CI 1.74–2.12] and in students in group 3 it was 3.14 [95% CI 2.98–3.30] (*p* < 0.001).

**Fig. 3:**
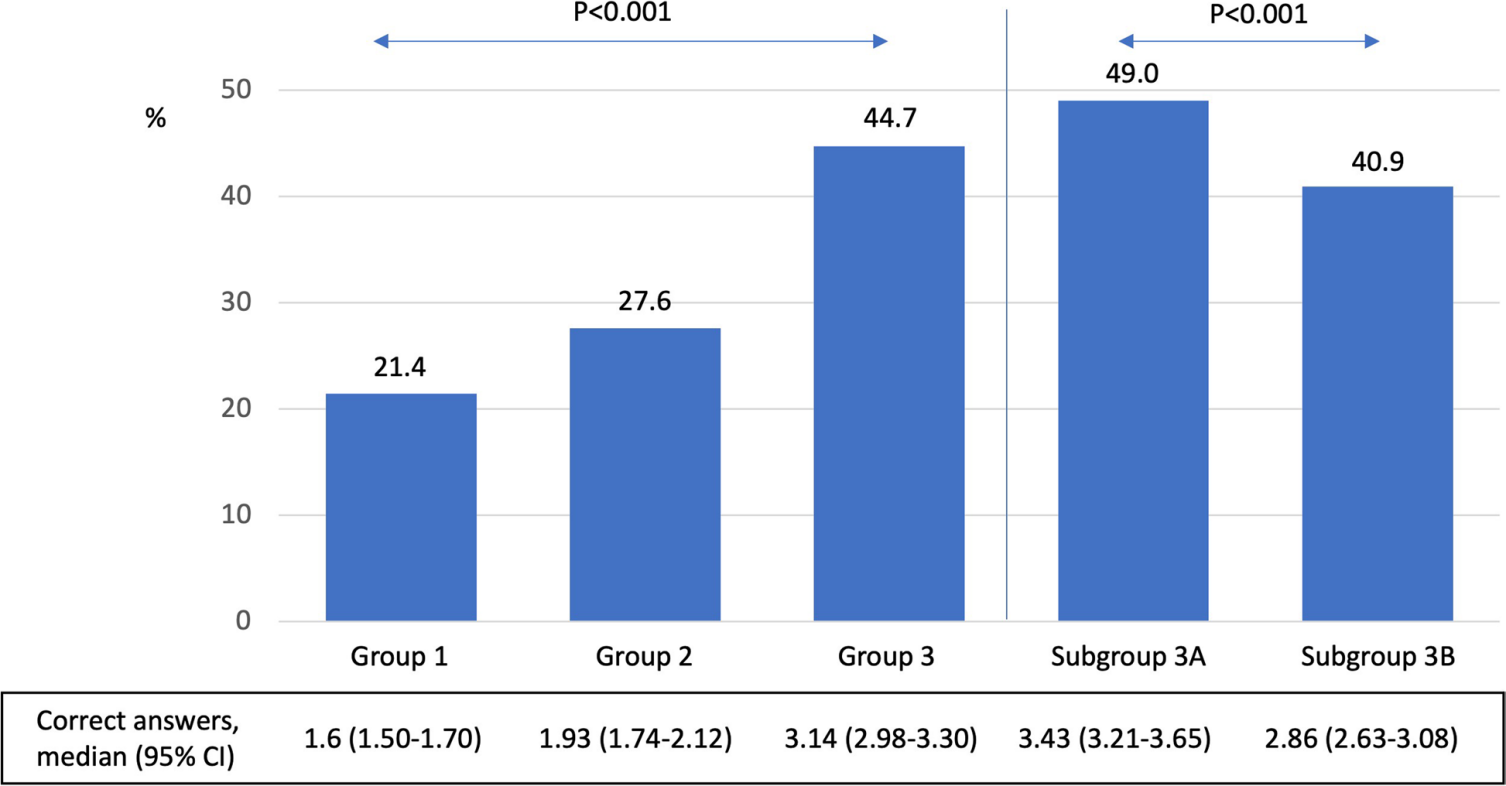
Correct answers between groups and subgroups of students

The percentage of correct answers for the group that had just received infectious disease training (group 3A) was 49% while the group that received it a year earlier (group 3B) it was 40.9%. The median number of correct answers given by students in group 3A was 3.43 [95% CI 3.21–3.65] and in students in group 3B it was 2.86 [95% CI 2.63–3.08] (*p* < 0.001). (See Fig. 3). A strong positive correlation was detected between year of training and number of correct answers (rho 0.95; *p* < 0.001).

## Discussion

Antibiotic resistance is a global public health concern, The World Health Organization defines the first objective in this area as being to improve the awareness and understanding of antibiotic resistance through effective communication, education and training of health workers [9].

In this study we describe the degree of awareness of medical students regarding antibiotic resistance, as well as the impact of training in the correct use of antibiotics. The high participation of the students (76.4%) demonstrates their interest in this problem. The perception and assessment of the problem is high from the first years of training onwards, but after training in infectious diseases, 99.3% of medical students consider that antibiotic resistance currently represents a Public Health problem and 98.4% think the problem of antibiotic resistance should be considered before starting an antibiotic prescription.

Training and education of future prescribers is one of the main interventions to improve the rational use of antibiotics. Training should begin in medical schools and continue throughout professional life. Studies have shown that doctors with limited knowledge about antibiotics tend to unnecessarily to prescribe more potent antibiotics and with a wider spectrum of action [10, 11].

Hecker et al., observed that the most frequent reasons for inappropriate antibiotic therapy are a duration of therapy that is longer than necessary, use of antibiotic for treatment of non-infectious or non-bacterial syndromes, treatment of colonization or contamination and redundant antimicrobial coverage [10].

In the first situation, even 90.5% of students in group 3 consider that there is no need to carry out long-term antibiotic treatment, but in the training questions section we found that only 14.3% of the students in this group were able to identify the appropriate duration of antibiotic therapy. Comparison of the findings with those of other studies confirms medical students’ a lack of confidence when identifying the correct duration of treatment or oral treatment alternatives [7]. The second most frequent cause of inappropriate antibiotic use was the treatment of patients with non-infectious or non-bacterial syndromes [12].

Regarding the duration of treatment, most of the students with complete training considered prolonged therapy unnecessary, compared to 76.5% of the students without training. This finding is relevant because it shows that future prescribers are more likely to modify their treatment approaches during their formative years. Modifying these attitudes in infectious disease physicians is often harder, despite clinical trials showing that the result does not differ. Recognition of undergraduate training as the imprinting phase to instill the concept of shorter-is-better could avoid future adverse effects, resistance to antibiotics, and reduce cost [13].

In our study, we observed a significant improvement in the ability of medical students to make an accurate diagnosis of infection after infectious disease training. Misdiagnosis has been found to be a leading cause of unnecessary treatments [14]. In the questions designed to evaluate the value of microbiological data, the impact of training is also relevant, although it has already begun after training in clinical microbiology (group 2). Finally, related to the impact of redundant antimicrobial coverage in inappropriate antibiotic use, we do not observe a significant improvement after training in infectious diseases. As in other points such as the choice of empirical antibiotic treatment, this result could be related to the high perception of students of these problems from the first years.

After training in infectious diseases, knowledge of the stewardship antibiotic program increases from 9.3% in medical students in group 1 to 52.2% in group 3. This situation contributes to a better perception of the problem of antibiotic resistance and a better and more rational use of these agents. However, these results represent another important point of improvement in medical student to accept the benefit of these programs. It is likely that improving the training at these points during medical schools could improve future prescribing habits [15].

In the second block, we also observed an improvement in results after training in infectious diseases. The average number of correct answers was 1.6 in group 1, 1.93 in group 2 and 3.14 in group 3, with statistically significant differences (*p* < 0.001). The average percentage of correct answers in students after training in infectious diseases was 52.7%. The work of Hecker et al. has shown that, in the hospital setting up to 50% of the prescriptions are inappropriate [10]. The impact of training in infectious diseases in our study was higher than that described by other authors [6, 7].

A final point is the partial loss of the formative impact over time. In our study we observed a median 9.7% decrease in correct answers (difference between subgroup 3A and subgroup 3B). This result could be explained by the fact that the students have forgotten what they have learned because they have not been subjected to tests, recall or spaced repetition [16].

Training in infectious diseases in medical school is an effective strategy to improve the use of antibiotics overall. However, as in other published studies, we observed a remarkable margin for improvement in training in infectious diseases and a rapid loss of knowledge one year after training [17]. Enhancing the training in the rational use of antibiotics in medical schools should represent an important stimulus for teachers to improve results, but this transformation will probably require changes in teaching methodology [18].

We must adopt pedagogical measures to stabilize the knowledge learned in infectious diseases in medical schools and, through continuous medical education, ensure adequate training for future prescribers. According to pedagogical studies, learning consists in reconstructing knowledge [19], that is, new knowledge is built on the existing foundations. Therefore, to add knowledge, you must have a solid foundation that allows the student to acquire more specialized knowledge of certain aspects [18]. In this context, Spanish students consider that the discussion of clinical cases and rotations in infectious diseases are among the most useful aspects of their medical degrees [20].

Several studies have investigated how to prevent the decline in students’ and professionals’ knowledge, and other forms of learning such as role-playing, teaching in workshops or e-learning can also be implemented and have been shown to improve students’ knowledge retention [18, 19].

The opinions-responses expressed by doctors and medical students should help us to propose changes when planning teaching in undergraduate and postgraduate training.

Another measure that could be introduced to improve the safety and rational use of antibiotics is the “P-drugs method”. P-drugs are those that are prescribed by each doctor regularly for a condition with which he/she is familiar. The list of medications varies between practitioners and depends on their knowledge and experience. The objective is for the prescriber to be familiar with the characteristics of each drug that it’s frequently used. This method, which is recommended by the WHO [21] and has successfully been applied in several countries, favors an adequate use of antibiotics and has shown good acceptance among students [22, 23].

Some limitations of this study are that, despite the high participation, it was carried out in a single center, participation in the survey depended on class attendance and an unvalidated questionnaire was used whose degree of discrimination in certain questions is difficult to assess.

## Conclusions

Our study shows that the training of medical students has a positive impact on their awareness of antibiotic resistance and favors a better use of antibiotics. This could contribute to mitigating one of the major global public health problems facing medicine today.

## Supplementary Information

**Additional file 1.**

## Data Availability

The datasets used and/or analyzed during the current study are available from the corresponding author upon reasonable request.

## Abbreviations

CM: Clinical Microbiology
ID: Infectious Diseases
