# Supplementary material for: Impact of Infectious Diseases training in the perception of antibiotic resistance and rational use of antibiotics among Spanish medical students – a cross-sectional study
Source: BMC Med Educ. 2022 Jul 15;22:550. doi: 10.1186/s12909-022-03580-8 (PMC9287956; doi:10.1186/s12909-022-03580-8)
Supplement: Supplementary file 1 — Additional file 1. [file 12909_2022_3580_MOESM1_ESM.docx]

*Supplementary material*

**Impact of Infectious Diseases training in the perception of antibiotic resistance and rational use of antibiotics among Spanish medical students. A cross-sectional study.**

*José Ramón Yuste^1,2^*, Andrés Blanco-Di Matteo*^1,2^*,Fernando Gruber*^1^*.

1.Division of Infectious Diseases, Clínica Universidad de Navarra, Pamplona, Spain

2.Department of Internal Medicine, Clínica Universidad de Navarra, Pamplona, Spain

**Corresponding author:**

José Ramón Yuste MD, PhD. Division of Infectious Diseases. Department of Internal Medicine. Clínica Universidad de Navarra, Pamplona, Spain. Phone: 00 34 948 255 400. Fax: 00 34 948 296 500.

E-mail: [jryuste@unav.es](mailto:jryuste@unav.es)

https://orcid.org/0000-0001-8502-9539

*Online Resource 1. Survey and global responses*

**First block**

Questions to know the student´s perception about diagnosis, bacterial resistance, and appropriate antibiotic use

| Recognition of the problem | | | | |
| --- | --- | --- | --- | --- |
| *Question 1*. Do you think antibiotic resistance is a public health problem? | | | | |
|  | Always  n (%) | Often  n (%) | Rarely  n (%) | Never  n (%) |
| Group 1 (n=508) | 337 (66.3) | 145 (28.4) | 24 (4.8) | 2 (0.5) |
| Group 2 (n=142) | 111 (78.1) | 26 (18.3) | 1 (0.7) | 4 (2.9) |
| Group 3 (n=294) | 257 (87.6) | 34 (11.7) | 1 (0.2) | 2 (0.5) |
| Subgroup 3A (n=151) | 134 (88.7) | 14 (9.3) | 1 (0.6) | 2 (1.4) |
| Subgroup 3B (n=143) | 123 (86.1) | 20 (13.9) | 0 | 0 |
|  | | | | |
| Microbiological diagnosis | | | | |
| *Question 2*. Do you think collect specimen for microbiological cultures before start empiric antibiotic is mandatory? | | | | |
|  | Always  n (%) | Often  n (%) | Rarely  n (%) | Never  n (%) |
| Group 1 (n=508) | 136 (26.8) | 212 (41.8) | 151 (29.6) | 9 (1.8) |
| Group 2 (n=142) | 54 (38) | 59 (41.6) | 20 (14.1) | 9 (6.3) |
| Group 3 (n=294) | 124 (42.3) | 141 (48) | 26 (8.7) | 3 (1) |
| Subgroup 3A (n=151) | 82 (55) | 59 (38.4) | 7 (4.7) | 3 (1.9) |
| Subgroup 3B (n=143) | 42 (29.3) | 82 (57.6) | 19 (13.1) | 0 |
|  | | | | |
| Empiric antibiotic treatment | | | | |
| Question 3. In a clinical stable patient with documented clinical infection, is it necessary to start empirical antibiotic treatment? | | | | |
|  | Always  n (%) | Often  n (%) | Rarely  n (%) | Never  n (%) |
| Group 1 (n=508) | 110 (21.6) | 287 (56.7) | 81 (15.8) | 30 (5.9) |
| Group 2 (n=142) | 27 (19.1) | 69 (48.7) | 30 (21.2) | 16 (11) |
| Group 3 (n=294) | 59 (20) | 164 (55.7) | 37 (12.7) | 34 (11.7) |
| Subgroup 3A (n=151) | 26 (17.2) | 64 (42.3) | 31 (20.6) | 30 (19.9) |
| Subgroup 3B (n=143) | 33 (23.7) | 100 (69.2) | 6 (4.2) | 4 (2.9) |
|  | | | | |
| *Question 4*. Before starting an empirical antibiotic treatment, we should consider the problem of antibiotic resistance? | | | | |
|  | Always  n (%) | Often  n (%) | Rarely  n (%) | Never  n (%) |
| Group 1 (n=508) | 384 (75.5) | 107 (20.9) | 16 (3.4) | 1 (0.2) |
| Group 2 (n=142) | 105 (73.9) | 31 (21.8) | 6 (4.3) | 0 |
| Group 3 (n=294) | 237 (81) | 52 (17.4) | 2 (0.6) | 3 (1) |
| Subgroup 3A (n=151) | 123 (81.6) | 24 (15.9) | 1 (0.6) | 3 (1.9) |
| Subgroup 3B (n=143) | 114 (79.7) | 28 (19.6) | 1 (0.7) | 0 |
|  | | | | |
| De-escalation therapy | | | | |
| *Question 5*. Do you think empirical antibiotic treatment should be adjusted according to microbiological data and clinical evolution? | | | | |
|  | Always  n (%) | Often  n (%) | Rarely  n (%) | Never  n (%) |
| Group 1 (n=508) | 404 (79.4) | 89 (17.5) | 14 (2.9) | 1 (0.2) |
| Group 2 (n=142) | 116 (81.7) | 21 (14.7) | 5 (3.6) | 0 |
| Group 3 (n=294) | 258 (88.1) | 29 (9.7) | 4 (1.2) | 3 (1) |
| Subgroup 3A (n=151) | 136 (90.1) | 10 (6.6) | 2 (1.4) | 3 (1.9) |
| Subgroup 3B (n=143) | 122 (86) | 19 (12.6) | 2 (1.4) | 0 |
|  | | | | |
| Combination therapy | | | | |
| *Question 6*. Do you think antibiotic combinations improve clinical results? | | | | |
|  | Always  n (%) | Often  n (%) | Rarely  n (%) | Never  n (%) |
| Group 1 (n=507) | 205 (40.3) | 270 (53.2) | 29 (5.8) | 3 (0.7) |
| Group 2 (n=142) | 61 (42.9) | 69 (48.6) | 11 (7.7) | 1 (0.8) |
| Group 3 (n=293) | 85 (29.1) | 164 (56.2) | 38 (12.8) | 6 (1.9) |
| Subgroup 3A (n=150) | 52 (28.4) | 72 (54.9) | 21 (13.4) | 5 (3.3) |
| Subgroup 3B (n=143) | 43 (30) | 83 (58.1) | 17 (11.9) | 0 |
|  | | | | |
| Prolonged duration | | | | |
| *Question 7*. Do you think prolonged duration of antibiotic treatment more than recommended by clinical guidelines improves clinical outcomes? | | | | |
|  | Always  n (%) | Often  n (%) | Rarely  n (%) | Never  n (%) |
| Group 1 (n=507) | 34 (6.9) | 81 (16.3) | 209 (40.9) | 183 (35.9) |
| Group 2 (n=142) | 8 (5.6) | 12 (7.5) | 81 (58) | 41 (28.9) |
| Group 3 (n=292) | 6 (2) | 22 (7.5) | 149 (51) | 115 (39.5) |
| Subgroup 3A (n=150) | 6 (3.9) | 8 (5.3) | 67 (44.4) | 69 (46.4) |
| Subgroup 3B (n=142) | 0 | 14 (9.8) | 82 (58) | 46 (32.2) |
|  | | | | |
| Appropriate route | | | | |
| *Question 8*. Conversion from parenteral to oral therapy of antibiotics with excellent bioavailability in clinically stable infected should be consider? | | | | |
|  | Always  n (%) | Often  n (%) | Rarely  n (%) | Never  n (%) |
| Group 1 (n=507) | 46 (9) | 199 (39.3) | 198 (39.1) | 64 (12.6) |
| Group 2 (n=142) | 24 (16.9) | 80 (56.3) | 29 (20.5) | 9 (6.3) |
| Group 3 (n=292) | 99 (34) | 146 (50.2) | 36 (12.3) | 11 (3.5) |
| Subgroup 3A (n=150) | 58 (38.1) | 71 (47.4) | 16 (11.2) | 5 (3.3) |
| Subgroup 3B (n=142) | 41 (28.7) | 75 (53.1) | 20 (14) | 6 (4.2) |
|  | | | | |
| Antibiotics for restricted use | | | | |
| *Question 9*. Do you think use of restricted drugs provide a clinical and microbiological benefit over first-line drugs? | | | | |
|  | Always  n (%) | Often  n (%) | Rarely  n (%) | Never  n (%) |
| Group 1 (n=507) | 85 (16.5) | 247 (48.5) | 155 (30.1) | 20 (4.9) |
| Group 2 (n=142) | 20 (14.1) | 69 (48.6) | 49 (34.5) | 4 (2.8) |
| Group 3 (n=292) | 26 (8.8) | 108 (37.1) | 144 (49.3) | 14 (4.8) |
| Subgroup 3A (n=150) | 17 (11.2) | 51 (34.5) | 70 (46.3) | 12 (8) |
| Subgroup 3B (n=142) | 9 (6.3) | 57 (39.9) | 74 (52.4) | 2 (1.4) |
|  | | | | |
| Antibiotic cost | | | | |
| *Question 10*. Do you think cost of antibiotic treatment should be considered before its prescription? | | | | |
|  | Always  n (%) | Often  n (%) | Rarely  n (%) | Never  n (%) |
| Group 1 (n=507) | 124 (24.2) | 216 (42.8) | 101 (19.9) | 66 (13.1) |
| Group 2 (n=141) | 50 (35) | 71 (50) | 12 (8.4) | 8 (6.6) |
| Group 3 (n=291) | 103 (35) | 145 (50.4) | 35 (11.9) | 8 (2.7) |
| Subgroup 3A (n=150) | 42 (27.8) | 79 (53) | 22 (14.5) | 7 (4.7) |
| Subgroup 3B (n=142) | 61 (43.3) | 66 (46.9) | 14 (9.1) | 1 (0.7) |
|  | | | | |
| Antibiotic stewardship program | | | | |
| *Question 11*. Do you know the Antibiotic Stewardship Program? | | | | |
|  | Yes  n (%) | No  n (%) |  |  |
| Group 1 (n=507) | 47 (9.3) | 460 (90.7) |  |  |
| Group 2 (n=141) | 13 (9.0) | 128 (91) |  |  |
| Group 3 (n=291) | 152 (52.2) | 139 (47.8) |  |  |
| Subgroup 3A (n=150) | 96 (63.8) | 54 (36.2) |  |  |
| Subgroup 3B (n=141) | 56 (41) | 85 (59) |  |  |
|  | | | | |
| Question 12. Do you think antibiotic stewardship program can improve medical training and clinical outcomes in the hospitals in which they are established? | | | | |
|  | Always  n (%) | Often  n (%) | Rarely  n (%) | Never  n (%) |
| Group 1 (n=507) | 305 (60.1) | 197 (38.9) | 0 (0) | 5 (1) |
| Group 2 (n=141) | 99 (70.4) | 42 (29.6) | 0 (0) | 0 (0) |
| Group 3 (n=291) | 203 (69.8) | 86 (29.7) | 0 (0) | 2 (0.5) |
| Subgroup 3A (n=150) | 101 (67.3) | 48 (31.8) | 0 (0) | 1 (0.9) |
| Subgroup 3B (n=141) | 104 (74) | 37 (26) | 0 (0) | 0 (0) |

**Second block**

**Questions to evaluate the knowledge in infectious diseases.**

| Relevance of the problem | |
| --- | --- |
| *Question 13*. Which of the following microorganisms does NOT currently represent a multiresistant problem?   - *Klebsiella pneumoniae* carbapenemases producer - Multi-resistant *Pseudomonas aeruginosa* - Penicillin resistant *Staphylococcus aureus* - *Enterococcus faecium* resistant to vancomycin | |
|  | Correct answer, n (%) |
| Group 1 (n=508) | 145 (28.6) |
| Group 2 (n=142) | 41 (28.9) |
| Group 3 (n=294) | 163 (55.4) |
| Subgroup 3A (n=151) | 111 (73.5) |
| Subgroup 3B (n=143) | 51 (35.7) |
|  | |
| Microbiological cultures | |
| *Question 14*. In a patient with septic shock with decreased level of consciousness and anuria, before starting antibiotic treatment, is mandatory the collection of:   - Urine culture - Sputum culture - Blood culture - None of the above. The priority is to start adequate antibiotic therapy | |
|  | Correct answer, n (%) |
| Group 1 (n=508) | 173 (34.1) |
| Group 2 (n=142) | 77 (54.2) |
| Group 3 (n=294) | 169 (57.5) |
| Subgroup 3A (n=151) | 92 (61) |
| Subgroup 3B (n=143) | 77 (53.8) |
|  | |
| Empiric antibiotic treatment | |
| *Question 15*. In a patient with a mild infection with isolation of microorganism sensitive to all the following antibiotics, what would be the first line?   - Amoxicillin - Amoxicillin clavulanic - Meropenem - Ciprofloxacin | |
|  | Correct answer, n (%) |
| Group 1 (n=508) | 163 (32.1) |
| Group 2 (n=142) | 47 (33.1) |
| Group 3 (n=294) | 89 (30.3) |
| Subgroup 3A (n=151) | 45 (29.8) |
| Subgroup 3B (n=143) | 44 (30.8) |
|  | |
| *Question 16*. What is the correct attitude in a patient with pharyngeal discomfort, rhinorrhea, conjunctivitis, fever, and involvement of the general condition, with physical exam just remarkable for an erythematous oropharynx?   - The use of antibiotics is necessary because it speeds up the patient's recovery - We will only consider an antibiotic if the patient is being treated with corticosteroids. - When in doubt, if the patient requests the prescription of an antibiotic, its administration is recommended - The use of antibiotics is not necessary | |
|  | Correct answer, n (%) |
| Group 1 (n=508) | 111 (21.9) |
| Group 2 (n=142) | 57 (40.1) |
| Group 3 (n=294) | 185 (62.9) |
| Subgroup 3A (n=151) | 97 (64.2) |
| Subgroup 3B (n=143) | 88 (61.5) |
|  | |
| Question 17. Asymptomatic bacteriuria requires treatment in:   - Diabetic patients - Patients with urinary catheter - Pregnant woman - All patients with a significant count (> 10^5^ CFU / mL) of a urine pathogen | |
|  | Correct answer, n (%) |
| Group 1 (n=508) | 98 (19.3) |
| Group 2 (n=142) | 23 (16.2) |
| Group 3 (n=294) | 219 (74.4) |
| Subgroup 3A (n=151) | 114 (74.5) |
| Subgroup 3B (n=143) | 105 (73.4) |
|  | |
| Appropriate route | |
| Question 18. In a stable patient hospitalized for an infectious disease with identification of sensitive microorganism and under treatment with levofloxacin. Do you consider that the intravenous route is more effective than the oral route?   - Yes - No | |
|  | Correct answer, n (%) |
| Group 1 (n=508) | 23 (4.5) |
| Group 2 (n=142) | 13 (9.2) |
| Group 3 (n=294) | 63 (21.4) |
| Subgroup 3A (n=151) | 39 (25.8) |
| Subgroup 3B (n=143) | 25 (17.5) |
|  | |
| Duration of therapy | |
| Question 19. The duration of treatment of a serious infection such as meningococcal meningitis is:   - 7 days - 14 days - 21 days - 28 days | |
|  | Correct answer, n (%) |
| Group 1 (n=508) | 57 (11.2) |
| Group 2 (n=142) | 18 (12.6) |
| Group 3 (n=294) | 42 (14.3) |
| Subgroup 3A (n=151) | 23 (15.2) |
| Subgroup 3B (n=143) | 19 (13.3) |
